# Trends and outcomes in diabetic ketoacidosis: A 10-year retrospective study at a Norwegian university hospital

**DOI:** 10.1371/journal.pone.0362629

**Published:** 2026-11-13

**Authors:** Sebastian Breivik Soleim, Kristian Løvås, Eirik Søfteland, Grethe Åstrøm Ueland

**Affiliations:** Department of Endocrinology, Haukeland University Hospital, Bergen, Norway; University of Colombo Faculty of Medicine, SRI LANKA

## Abstract

**Aims:**

To describe trends in diabetic ketoacidosis admissions and examine factors associated with repeated admissions and mortality among adults living with type 1 diabetes.

**Materials and methods:**

Diabetic ketoacidosis admissions among adults at a Norwegian university hospital between 1 August 2012 and 31 August 2022 were retrospectively reviewed using electronic medical records.

**Results:**

A total of 178 participants with 306 admissions were included. Median [IQR] age at admission was 34 years [22–51]. Median HbA1c was 88 mmol/mol [72–105]. Diabetic ketoacidosis admission frequency decreased by an estimated 6.9% annually (RR 0.931; 95% CI 0.896–0.968; p < 0.001) over the 10-year period. 40 participants (22.5%) had more than one admission during the study period. Psychiatric illness (aOR 2.827; 95% CI 1.070–7.468; p = 0.036) and receipt of disability benefits (aOR 4.941; 95% CI 1.576–15.490; p = 0.006) were associated with experiencing more than one lifetime admission. Prior diabetic ketoacidosis, suspected treatment non-adherence and psychiatric illness were associated with higher readmission rates during follow-up. In-hospital mortality was 1.6%, and 5-year cumulative mortality was 14.7%. Alcohol or substance misuse (HR 2.499; 95% CI: 1.191–5.245; p = 0.015) and higher age at the index admission (HR 1.874; 95% CI: 1.511–2.324; p < 0.001, per 10 years) were associated with mortality in the follow-up period.

**Conclusions:**

Diabetic ketoacidosis admissions declined substantially over the 10-year study period despite growth in the hospital catchment area population, suggesting that diabetes care has improved. Repeated admissions were common and associated with psychosocial vulnerability. In-hospital mortality was low, but five-year mortality was considerable, supporting targeted follow-up after an episode of diabetic ketoacidosis.

## 1. Introduction

Diabetic ketoacidosis (DKA) remains a serious complication of type 1 diabetes despite substantial advances in diabetes care, including continuous glucose monitoring, insulin pumps and automated insulin delivery [1]. Recent studies have reported increasing DKA admission rates in the UK and USA [2,3], whereas declining rates have been observed in Denmark [4]. These contrasting trends highlight the importance of examining how DKA admission trends have evolved in different healthcare settings during a period of changing diabetes management.

The clinical burden of DKA extends beyond the acute admission. Although in-hospital mortality from ketoacidosis remains low in most high-resource settings [1], DKA is associated with increased long-term mortality, and repeated episodes account for a substantial portion of admissions and may be a marker of particular vulnerability [5,6]. Understanding the clinical and psychosocial characteristics associated with repeated admissions and mortality may help identify patients who could benefit from targeted follow-up and prevention strategies.

To understand the impact and burden of DKA, admission trends and their development over time need to be examined along with the circumstances of individual admissions and subsequent patient outcomes. Detailed clinical cohorts can provide this complementary perspective. We therefore examined trends in ketoacidosis admissions at a Norwegian university hospital from 2012 to 2022 and characterised the clinical presentation and subsequent outcomes. We also assessed factors associated with recurrent admissions and death.

## 2. Materials and methods

We conducted a retrospective cohort study of people with type 1 diabetes admitted with ketoacidosis to the adult service at Haukeland University Hospital in Bergen, between 1 August 2012 and 31 August 2022. The hospital’s catchment area comprised approximately 288 000 residents, 232 000 of whom were 16 or older, in 2022 [7,8]. Applying the national type 1 diabetes prevalence estimate of 0.72% [9] among adults aged 18 years or older to the catchment population aged 16 or older gives a rough estimate of approximately 1700 people with type 1 diabetes in 2022. Following approval from the Regional Committee for Medical and Health Research Ethics (REK no. 511 576), eligible persons were identified through an index search in the hospital electronic medical records.

### 2.1. Study population

The search of all discharges with an ICD-10 diagnosis of either E10.0 (type 1 diabetes with coma) or E10.1 (type 1 diabetes with ketoacidosis) yielded 330 unique persons with 504 admissions. Participants were informed by letter and given the opportunity to opt out of participation. Participants were excluded if they lived abroad, withdrew consent or were under the age of 18 at the time of inclusion in 2023 (n = 27). Following review of diabetes classification and evaluation of clinical and biochemical findings, 178 persons and 306 admissions were included. The American Diabetes Association diagnostic criteria for type 1 diabetes and diabetic ketoacidosis [1,10] were employed. Ketone values were unavailable in 111 admissions, and five additional admissions had documented values below the diagnostic threshold. Such admissions were retained only if individual chart review, the treating endocrinologist’s assessment, the clinical presentation and response to DKA treatment were consistent with a DKA diagnosis. Admissions with a plausible alternative explanation for acidosis were excluded.

### 2.2. Data collection

Hospital medical records for individual participants were systematically reviewed by one investigator (SBS) between January and December 2024, with access to identifiable patient information. Clinician-noted demographics, lifestyle factors, diabetes history, complications, somatic and psychiatric comorbidities, history of alcohol or substance misuse, treatment adherence, insulin regimen, presenting clinical parameters, treatment initiated at admission, and short- and long-term outcomes were recorded. The electronic medical records are synchronised with the Norwegian National Population Register, yielding date but not cause of death for deceased participants. The cutoff time for admission ascertainment was August 31^st^, 2022, and for death November 26^th^, 2024.

### 2.3. Definitions

The most recent diabetes classification recorded in the electronic medical records was used, and C-peptide and antibody levels were not checked. Psychiatric comorbidities were registered in the case of psychiatric illnesses that had required pharmacological or psychological therapy, including anxiety, depression, psychosis and eating disorders, registered in the hospital electronic medical records at the index admission. Alcohol or substance misuse was defined as a previously documented or currently suspected overconsumption of alcohol or psychoactive substances at the index admission. Cognitive impairment was registered if it had been noted in the electronic medical records at any point prior to the index admission, regardless of cause. Suspected treatment non-adherence was defined as several documented missed follow-up appointments or known or suspected medication non-compliance, based on records available at the index admission. Receipt of disability benefits (uføretrygd), irrespective of cause, was noted when explicitly documented at the index admission. The presumed cause of ketoacidosis was estimated by the treating physician and/or by the reviewer based on electronic medical record entries. Persons with and without known diabetes mellitus prior to admission were examined separately and are referred to as having pre-existing or new-onset diabetes mellitus.

The ketone criterion was a capillary blood ketone concentration ≥ 3 mmol/L or urine ketones ≥ 2 + . Time to biochemical normalisation was defined as time from first recorded arterial blood gas analysis until the first arterial blood gas analysis with a recorded bicarbonate ≥18 mmol/L. For mortality analyses, time was defined as months from the index admission in the study period to death or censoring.

### 2.4. Outcomes

Outcome measures included temporal trends in DKA admission counts, time to biochemical normalisation, length of hospital stay, recurrent DKA admissions, in-hospital mortality and all-cause mortality following the index admission.

### 2.5. Statistical analyses

Continuous variables are reported as medians with interquartile ranges (IQR), and categorical variables as n (% of available observations). Laboratory measurements are reported in SI units. Effect estimates are reported as rate ratios (RR) for Poisson and negative binomial regressions, odds ratios (OR) for logistic regression, hazard ratios (HR) for Cox regression, and ratios of expected time for gamma regression, all with 95% confidence intervals. A two-sided p-value <0.05 was considered significant.

Covariates for each multivariable analysis were selected on clinical grounds and entered simultaneously. Participant-level models used characteristics present at the index admission, whereas admission-level models used characteristics recorded for each individual admission. Sensitivity analyses restricted to admissions meeting the documented ketone criterion were performed to assess robustness of findings.

Hospital monthly admission counts were analysed using Poisson regression with a log link and calendar time in years. DKA recurrence analyses included patients with previously known diabetes. A binary logistic regression model examined risk of multiple lifetime admissions, while a Cox regression model was used to examine time from discharge to first recurrent admission in the study period, censoring at death or August 31^st^ 2022. In addition, a negative binomial regression analysis was performed to model all subsequent readmissions using log-transformed person-time from index discharge to death or August 31^st^ 2022 as an offset. The Cox and negative binomial regression analyses of recurrence were restricted to participants who survived their index admission.

Five-year mortality was estimated with a Kaplan-Meier analysis from the first observed admission. Cox regression of all-cause mortality used all available follow-up information until the 26^th^ of November 2024. An additional Cox model examined recurrence status as a time-varying covariate for mortality.

Time to biochemical normalisation and length of stay were analysed using gamma regression with a log link and generalised estimating equations to account for repeated admissions.

Covariate sets, missingness documentation, model diagnostics and sensitivity analyses are detailed in the Supplementary appendix. For the recurrence analyses, multiple imputations were performed, while the rest of the analyses used complete cases. All analyses were performed using IBM SPSS Statistics v31.

## 3. Results

In total, 178 unique participants with 306 admissions were included. Baseline characteristics and diabetes history are summarised in Table 1.

**Table 1:** Baseline characteristics and diabetes history at first observed DKA admission.

Characteristic	n (%) or median [IQR]		
Diagnosis	Pre-existing T1D (n = 148)	New-onset T1D (n = 30)	Total (n = 178)
Female sex	69 (46.6%)	13 (43.3%)	82 (46.1%)
Age at first admission, years	36 [21-52]	32 [23-47]	34 [22–51]
Smoking (current or former)	68 (45.9%)	12 (40%)	80 (44.9%)
Alcohol or substance misuse (current or former)	30 (20.3%)	2 (6.7%)	32 (18.0%)
Psychiatric illness	29 (19.6%)	3 (10%)	32 (18.0%)
Cognitive impairment	11 (7.4%)	1 (3.3%)	12 (6.7%)
Receiving disability benefits	26 (19.5%)	0 (0%)	26 (16.5%)
Treated hypertension	37 (25.0%)	0 (0%)	37 (20.8%)
Treated dyslipidaemia	35 (23.6%)	0 (0%)	35 (19.7%)
Coronary artery disease	14 (9.5%)	0 (0%)	14 (7.9%)
Peripheral arterial disease	10 (6.8%)	0 (0%)	10 (5.6%)
BMI ≥ 25 kg/m^2^	44 (38.9%)	13 (54.2%)	57 (41.6%)
Retinopathy	47 (32.0%)	0 (0%)	47 (26.6%)
Nephropathy	18 (12.2%)	0 (0%)	18 (10.2%)
Neuropathy	18 (12.2%)	0 (0%)	18 (10.2%)
Suspected treatment non-adherence	44 (29.7%)	0 (0%)	44 (24.7%)
Follow-up by diabetes specialist	116 (81.1%)	0 (0%)	116 (67.1%)

Characteristics refer to the first observed DKA admission. Percentages are calculated from the available observations. Denominators with decreased availability for pre-existing diabetes, new-onset diabetes and the total cohort, respectively, were 133, 25 and 158 for disability benefits; 113, 24 and 137 for BMI; 143, 30 and 173 for specialist follow-up; and 147, 30 and 177 for each microvascular complication. T1D = type 1 diabetes mellitus; BMI = Body mass index.

### 3.1. Clinical features

Clinical features during the admission are summarised in Table 2 and Fig 1. The most common identified precipitating cause was forgotten or voluntarily omitted insulin dose(s). Notably, a history of psychiatric illness was present in 40% of admissions in which insulin had been forgotten or intentionally omitted. In 83 admissions (30.1% of admissions among participants with pre-existing T1D) the patient was using an insulin pump, and 24 admissions were due to pump failure. In three of the admissions due to pump failure, the participant was using an automated insulin delivery device.

**Table 2:** Clinical characteristics, treatment and outcomes of DKA admissions.

Characteristic	n (%) or median [IQR]		
Diagnosed T1D prior to admission	Pre-existing (n = 276)	New-onset (n = 30)	Total (n = 306)
**Treatment regimen**			
Insulin pump use	83 (30.1%)	0 (0%)	83 (27.1%)
AID type insulin pump	6 (2.2%)	0	6 (2%)
Use of continuous glucose monitoring	51 (18.5%)	0	51 (16.7%)
**Presumed cause**			
Infection	63 (22.8%)	4 (13.3%)	67 (21.9%)
Forgotten or omitted insulin	91 (33.0%)	0	91 (29.7%)
Pump failure any type	24 (8.7%)	0	24 (7.8%)
Alcohol or other intoxication	23 (8.3%)	0	23 (7.5%)
Unknown or other	75 (27.2%)	26 (86.7%)	101 (33.0%)
**Biochemistry**			
HbA1c, mmol/mol	86 [71–103], n = 276	109.5 [93–133], n = 30	88 [72–105], n = 306
GCS (n = 299)*			
Mild (13–15)	252 (93.3%)	28 (96.6%)	280 (93.6%)
Moderate (9–12)	10 (3.7%)	0 (0%)	10 (3.3%)
Severe (3–8)	8 (3.0%)	1 (3.4%)	9 (3.0%)
Severity* mild/moderate/severe			
Mild	34 (12.5%)	4 (13.3%)	38 (12.6%)
Moderate	79 (29.2%)	10 (33.3%)	89 (29.6%)
Severe	158 (58.3%)	16 (53.3%)	174 (57.8%)
**Level of care after ED ward/IMCU/ICU**	24/122/129 (8.7%/44.4%/46.9%)	2/18/10 (6.7%/60.0%/33.3%)	26/140/139 (8.5%/45.9%/45.6%)
**Outcomes**			
Time until HCO_3_^-^ ≥ 18 mmol/L (hours)	16 [9.5–25.0], n = 249	15.75 [7.9–23.4], n = 30	16 [9.0–25.0], n = 279
Hospital length of stay (hours)	85.5 [54.5–132.0], n = 271	178.75 [143.3–221.4], n = 30	93.5 [58.8–148.0], n = 301
Re-admitted within 30 days of discharge (from any cause)	50 (18.5%)	0	50 (16.6%)
Deceased within 30 days of discharge (from any cause)	2 (0.7%)	0	2 (0.7%)
Deceased during admission	5 (1.8%)	0	5 (1.6%)

Data are presented as n (%) or median [IQR = Interquartile range]. Percentages are calculated from available observations. Available denominators for pre-existing diabetes, new-onset diabetes and all admissions, respectively, were 270, 29 and 299 for GCS, 271, 30 and 301 for DKA severity, and 275, 30 and 305 for level of care. T1D, Type 1 Diabetes Mellitus; AID, Automated Insulin Delivery; GCS, Glasgow Coma Scale; ED, Emergency Department; IMCU, Intermediate-Care Unit; ICU, Intensive Care Unit; *According to ADA severity criteria. Three patients admitted after cardiac arrest and return of spontaneous circulation were excluded. American Diabetes Association criteria for severity were applied (Severity of DKA according to ADA criteria: mild (pH 7.25–7.30 or HCO_3_^−^ 15–18 mmol/L), moderate (pH 7.00–7.24 or HCO_3_^−^ 10–14.9 mmol/L), severe (pH < 7.00 or HCO_3_^−^ < 10 mmol/L)) [1].

**Fig 1:**
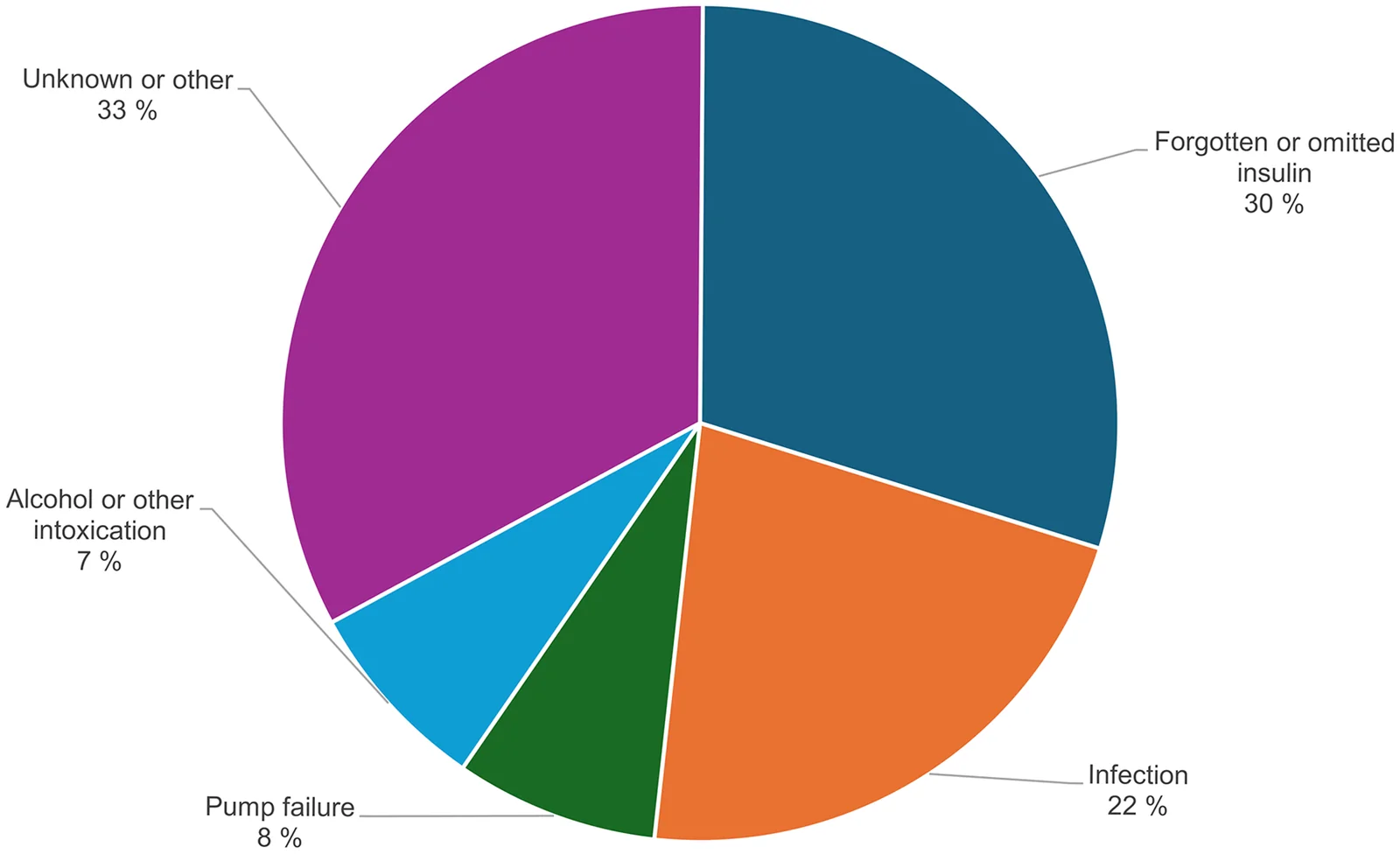
Precipitating causes of diabetic ketoacidosis. Distribution of presumed precipitating causes across 306 diabetic ketoacidosis admissions among 178 participants. Percentages were calculated using all 306 admissions and rounded for presentation.

### 3.2. Biochemistry and treatment

Median HbA1c at admission was 88 mmol/mol (IQR 72–105). In only 10 admissions (3.3%) HbA1c was 53 mmol/mol or lower. Using a 300 mOsm/kg cutoff, concomitant hyperosmolality was present in 44% of admissions. 58% of ketoacidoses were characterised as severe.

In the majority of admissions, the diagnosis was recognised in the emergency room and treatment with isotonic fluids (98%) and insulin (96%) was initiated according to local treatment guidelines. Hospital guidelines required potassium infusion prior to insulin when serum potassium was ≤ 5 mmol/L, but this was only initiated in 55% of eligible admissions. For biochemical parameters at admission, see S1 Appendix, Table S7.

### 3.3. Temporal trend in DKA admissions

Mean monthly DKA admission counts decreased from 3.20 in 2012 to 1.875 in 2022. In a Poisson regression, the decline was estimated at 6.9% per year (RR 0.931; 95% CI 0.896–0.968; p < 0.001), corresponding to a model-estimated reduction of approximately 51% over the 10-year follow-up period (Fig 2).

**Fig 2:**
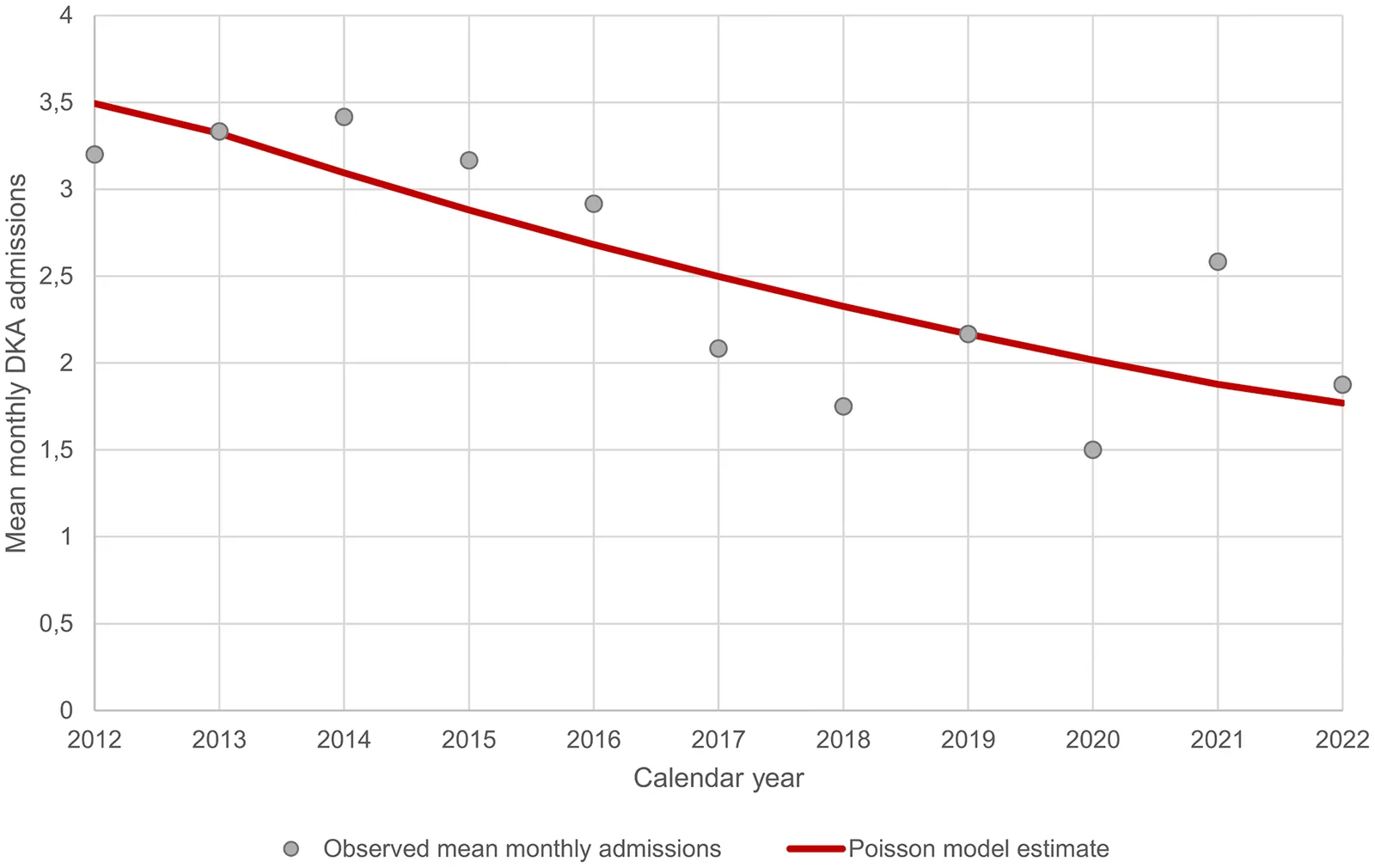
Temporal trend in diabetic ketoacidosis admissions. Grey circles show the observed mean monthly number of admissions in each calendar year. Data for 2012 cover August-December, and data for 2022 cover January-August. The red line shows the fitted trend from Poisson regression using all 121 monthly admission counts. The estimated annual rate ratio was 0.931 (95% CI 0.896-0.968; p < 0.001). DKA, diabetic ketoacidosis.

### 3.4. Time to biochemical normalisation

Each successive calendar year was associated with a 2.8% shorter time until resolution (p = 0.025) for a cumulative relative decrease of 24% during the 10-year period. Male sex, a higher pH and higher glucose at admission were associated with shorter time to resolution (S1 Appendix, Table S5A).

### 3.5. Length of hospital stay

New-onset diabetes and macrovascular disease were associated with 151% and 87% longer hospital stays. For each successive calendar year, length of stay decreased by 3.7% (S1 Appendix, Table S5B).

### 3.6. Recurrent admissions

Twenty-two percent of the participants had more than one admission during the 10-year follow-up period, and this group accounted for 55% of all admissions. A small subset of five persons (2.8%) had six or more episodes; one experienced 24 admissions. Among the 30 participants in whom a ketoacidosis admission marked the debut of their type 1 diabetes, two were admitted twice with ketoacidosis, and two were admitted thrice in the study period.

Among 148 participants with previously known diabetes, 63 had more than one DKA admission at any point prior to or in the study period. In a multivariable binary logistic regression, psychiatric illness (aOR 2.827; 95% CI 1.070–7.468; p = 0.036) and receipt of disability benefits (aOR 4.941; 95% CI 1.576–15.490; p = 0.006) were associated with experiencing more than one lifetime admission, while higher age at the index admission in the study period was associated with decreased odds (aOR 0.727; 95% CI 0.565–0.935; p = 0.013, per 10 years of age) (Fig 3A).

**Fig 3:**
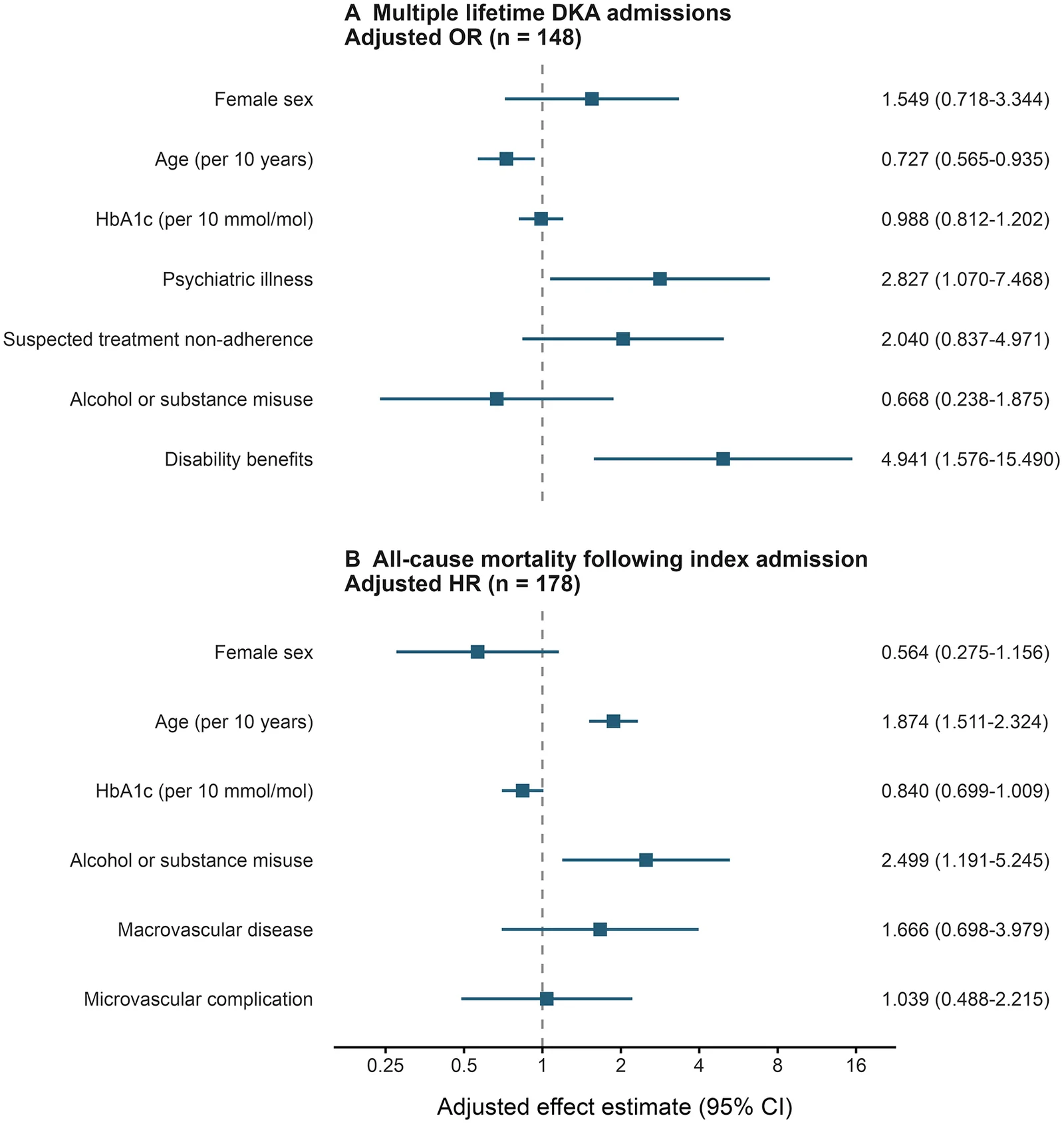
Factors associated with multiple lifetime diabetic ketoacidosis admissions and all-cause mortality. (A) Adjusted odds ratios from binary logistic regression for at least two versus one documented lifetime diabetic ketoacidosis admission among participants with pre-existing type 1 diabetes (n = 148). Estimates were pooled across 50 imputed datasets. (B) Adjusted hazard ratios from Cox regression for all-cause mortality from the first observed admission through 26 November 2024 (n = 178). Covariates were assessed at the first observed admission. Squares indicate point estimates, and horizontal lines indicate 95% confidence intervals. The horizontal axis uses a logarithmic scale. Age and HbA1c effects are expressed per 10 years and 10 mmol/mol, respectively. DKA, diabetic ketoacidosis; OR, odds ratio; HR, hazard ratio; CI, confidence interval; HbA1c, glycated haemoglobin.

Of the 143 participants with previously known diabetes who survived their index admission, 36 had a readmission in the study period, accounting for 122 readmissions in total. A secondary analysis with a Cox regression model analysing time to first readmission in the study period showed that documented DKA prior to the index admission was associated with a higher hazard of first readmission (HR 3.058; 95% CI 1.469–6.365; p = 0.003). A negative binomial model accounting for all subsequent DKA admissions in the study period showed that higher rates of recurrence within the 10-year period were associated with having experienced a previous episode (RR 3.755; 95% CI 1.790–7.877; p < 0.001), as well as with suspected treatment non-adherence (RR 4.402; 95% CI 1.835–10.570, p < 0.001) and psychiatric illness (RR 2.643; 95% CI 1.096–6.373; p = 0.030). No clear association with index-admission HbA1c was observed. Model results are reported in full in the S1 Appendix, Tables S3A and S3B.

### 3.7. Short-term outcomes and mortality

Following 50 (16.6%) admissions, the participant was readmitted for any cause within 30 days of discharge. Five admissions had a fatal outcome: in four, active treatment was withdrawn; in three due to anoxic brain injury caused by pre-hospital cardiac arrest and in one due to an inoperable, unrelated condition. In the final fatal admission, cardiac arrest occurred immediately upon arrival to the emergency room, before treatment could be initiated.

A Kaplan-Meier analysis showed a 5-year mortality from the index admission of 14.7% (95% CI 10.2–21.0) (Fig 4; S1 Appendix, Table S4A). Among the 36 participants who died during the full follow-up period, median age at death was 59.0 years (IQR 50.8–71.9). The results from a multivariable Cox regression model are shown in Fig 3B. A history of alcohol or substance misuse was associated with a 2.5-fold higher hazard of death in the follow-up period (HR 2.5; 95% CI 1.19–5.25; p = 0.015). For every 10 years higher age at the index admission, the hazard of death increased by 87% (HR 1.87; 95% CI 1.51–2.32; p < 0.001) (S1 Appendix, Table S4B). No clear associations with mortality were observed for index-admission HbA1c, micro- or macrovascular complications or recurrence (S1 Appendix, Tables S4B and S4C).

**Fig 4:**
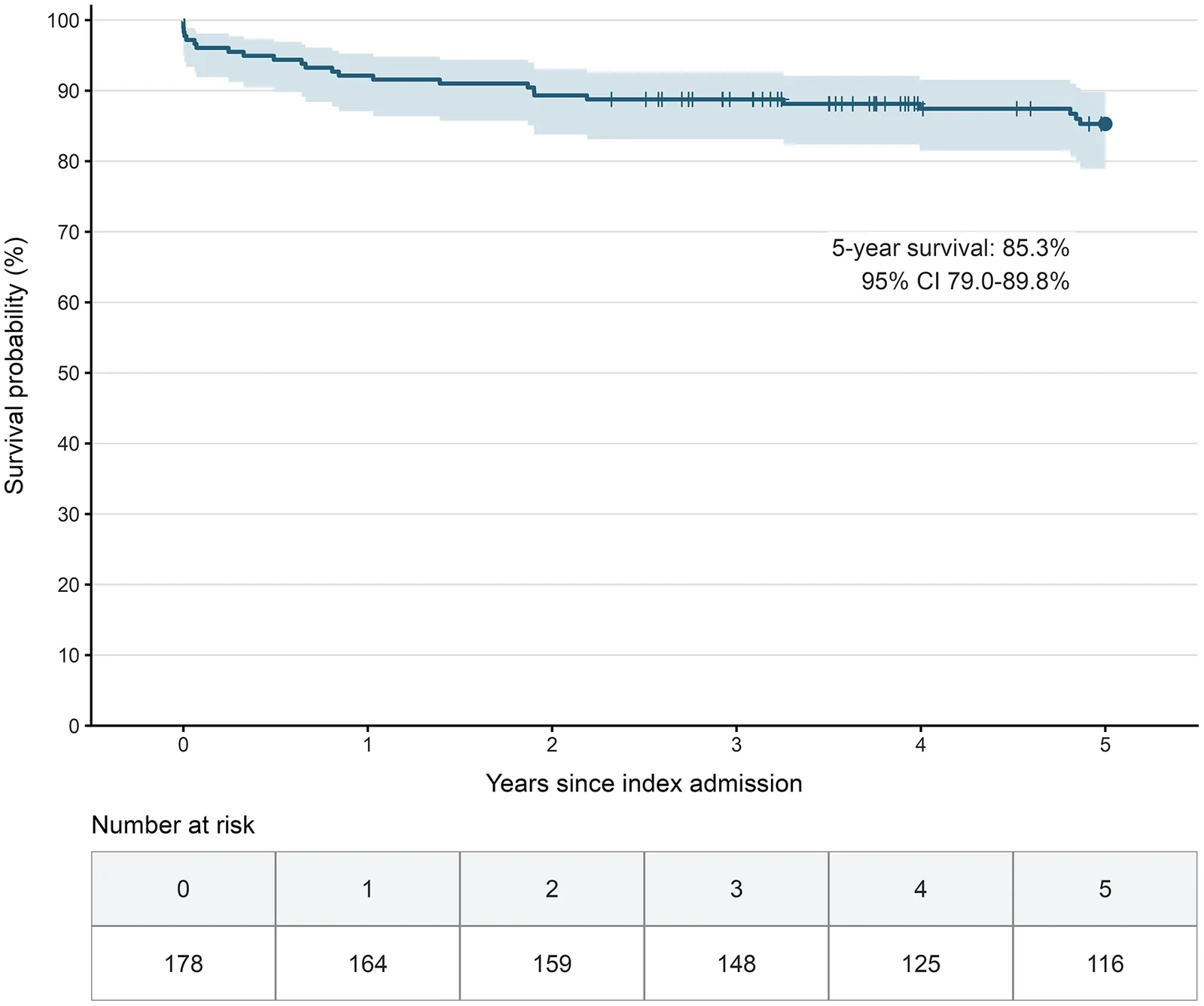
Five-year survival following the first observed diabetic ketoacidosis admission. Kaplan-Meier estimate of survival from the first observed diabetic ketoacidosis admission during the study period (n = 178). Shading indicates 95% confidence intervals, and tick marks indicate censored observations. The table gives the number at risk at yearly intervals. Estimated five-year survival was 85.3% (95% CI 79.0-89.8%). CI, confidence interval.

## 4. Discussion

Among key findings of the present study was a substantial cumulative decline in hospital DKA admissions. Psychiatric illness and receipt of disability benefits were associated with multiple lifetime admissions, while prior DKA, psychiatric illness and suspected treatment non-adherence were associated with higher readmission rates during the study period. Most participants presented with severe acidosis, indicating that emergency care is often sought relatively late in the disease course. In-hospital mortality was low, but 5-year mortality after the index admission was considerable. A higher age was predictably associated with increased mortality, as was alcohol or substance misuse.

The estimated 51% cumulative reduction in DKA admission frequency observed over the study period possibly reflects improved diabetes management, increased adoption of advanced treatment technologies, and better education for affected persons. Interestingly, the findings contrast trends reported from Finland, the UK and the US [2,3,11], but are in line with findings in Denmark [4]. The difference from our findings may be influenced by regional and temporal differences in health care system factors and access to advanced treatment technologies.

Increasing use of continuous glucose monitoring (CGM) may have contributed to the observed decline in hospital admission counts. Norwegian registry data document a substantial increase in use between 2012 and 2020, and initiation was associated with improved glycaemic control [12]. Among patients attending our clinic, CGM use increased from 5% at the start of the study period to 15% in 2016 and 81% by 2022, coinciding with the most pronounced decline in DKA admissions. These findings are consistent with French reports of reduced DKA admissions following initiation of flash glucose monitoring [13], although its contribution to the decline observed in our cohort cannot be quantified.

We are not aware of systematic changes in local admission thresholds or referral practices during the study period that could otherwise explain the trend. The decline occurred despite growth in the catchment area population in the relevant age categories, from 202,000 in 2012 to 231,760 in 2022, or an increase of 14.7% [7,8]. Assuming a stable type 1 diabetes prevalence, this would increase the population at risk from around 1500 to around 1700 from 2012 to 2022 [9].

Pump failure, whether due to user or equipment error, was a less frequent precipitating cause than reported in a German cohort [14]. Very few admissions were caused by failure of an automated insulin delivery device operating in closed-loop mode. Among patients included in our clinic annual reports, insulin pump use increased from 31% to 46% between 2016 and 2022, although registry coverage was incomplete until 2018. Observational studies have reported lower DKA rates following initiation of insulin pump therapy [15,16]. A formal analysis of their use was not feasible in this study, as adoption of this technology was only widespread in the final years of inclusion. The low proportion of automated insulin delivery device use among admitted persons does not suggest a major safety concern, but the small number of admissions and lack of a reliable population denominator limit conclusions about device-specific DKA risk and safety.

Among those with pre-existing diabetes, concurrent infection and missed insulin doses were the most commonly documented precipitating causes, in line with findings from other cohorts [1]. Notably, psychiatric comorbidity was disproportionately common in admissions where omitted or forgotten insulin administration contributed. While deliberate insulin restriction for weight control or as a form of self-harm cannot be confirmed, our findings align with earlier studies linking psychosocial vulnerability to an increased risk of ketoacidosis recurrence [6,17,18] and may in part explain the increased mortality seen in people with type 1 diabetes and eating disorders [19].

Glycaemic control at admission was generally poor, and almost no participants had HbA1c in the recommended range. Median HbA1c was much higher than in the total type 1 diabetes population in Norway, for whom median HbA1c declined to 57 mmol/mol in this period [20]. Although causality cannot be inferred, the marked difference supports the well-documented assumption that poor glycaemic control is a significant risk factor for ketoacidosis [1,21].

Repeated admissions were concentrated in a subgroup of participants, consistent with previous studies [22]. The associations with psychiatric illness and receipt of disability benefits indicate that attention to psychosocial vulnerabilities is warranted alongside the traditional approach of glucose monitoring and insulin treatment [23–25]. Post-DKA care should include assessment of mental health and substance use and reinforcement of diabetes self-management education. For pump users, this should include a clear contingency plan for recognising and managing interruptions to insulin delivery [1].

Time until resolution decreased by 2.8% per calendar year although official treatment protocols have remained the same, which may reflect improved adherence to local treatment guidelines. In line with this, length of stay decreased by 3.7% for each successive calendar year.

Incomplete adherence to potassium protocols highlights a potential area for improvement in DKA management. Hypokalaemia during treatment was not systematically recorded, but has been shown to be associated with increased in-hospital mortality [1].

In-hospital mortality was low after excluding those who experienced pre-hospital cardiac arrest. However, five-year all-cause mortality after the person’s first admission in the study period was high, in line with the findings from other cohorts in Scotland and New Zealand [5,6] and a large cohort of patients with hyperglycaemic crises in Taiwan [26].

Older age and alcohol or substance misuse were associated with higher all-cause mortality. The association with substance misuse may reflect the challenges of managing type 1 diabetes in the context of psychosocial vulnerability. Intoxication was a common contributing factor to ketoacidosis admission, highlighting the difficulty with maintaining advanced insulin therapy while under the influence of alcohol or psychoactive substances and suggesting that acute metabolic complications may be a contributing factor to the increased mortality in this group. Although a point estimate indicated higher mortality following recurrent DKA, the confidence interval was wide and results did not reach significance.

The substantial mortality and relatively young age at death may indicate that a ketoacidosis admission is a significant risk factor for premature death. Only all-cause mortality was examined, but infectious and cardiovascular disease have been found to be common causes in similar cohorts [5]. The extent to which acute metabolic complications contribute has not been fully elucidated. It is also possible that a ketoacidosis admission is an indicator of decreased health and ability to maintain self-care and thus a marker of increased risk of death from other causes.

### 4.1. Limitations

The major limitation of the study is the retrospective design, which relies on the accuracy and completeness of electronic medical records. Some records lacked information on disability benefits, time to biochemical normalisation or ketone measurements. Multiple imputations and sensitivity analyses addressing these gaps generally supported the main clinical findings, although some effect estimates varied or were less precise. For admission trends, comparisons between the full cohort and the admissions meeting the documented ketone criterion were limited by changes in documentation practices over time. Detailed sensitivity results are provided in the Supplementary appendix, Sections 1–6. No external control group was included, so comparative analyses with the broader diabetes population are limited. Annual local type 1 diabetes population counts were unavailable, precluding calculation of population-based DKA admission rates.

## 5. Conclusion

In conclusion, diabetic ketoacidosis admission counts declined markedly between 2012 and 2022 despite a probable increase in the number at risk in the catchment area of as much as 15%, likely reflecting improvements in diabetes care. Most participants presented with severe acidosis. Poor glycaemic control is common in those admitted for ketoacidosis. Psychiatric illness and receipt of disability benefits were associated with higher odds of multiple lifetime ketoacidosis admissions. Although in-hospital mortality was low, all-cause mortality following an admission was considerable, especially among older participants and persons with alcohol or substance misuse.

These findings highlight the need for targeted prevention strategies, long-term risk monitoring, and tailored support for vulnerable persons at risk of severe disease trajectories.

## Supporting information

S1 Appendix Supplementary methods and results. (DOCX)
