# Supplementary material for: Trends and outcomes in diabetic ketoacidosis: A 10-year retrospective study at a Norwegian university hospital
Source: PLoS One. 2026 Nov 13;21(11):e0362629. doi: 10.1371/journal.pone.0362629 (PMC13766292; doi:10.1371/journal.pone.0362629)
Supplement: Supplementary file 1 [file pone.0362629.s001.docx]

**Section 1: Ketone documentation and definition of the biochemical sensitivity cohort**

**1.1 Ketone measurement missingness**

The availability of ketone values was much better in the latter years of the study in correlation with the switch to capillary ketone measurements and the full implementation of electronic charting. Consequently, the absence of an available ketone value did not necessarily indicate that ketones had not been measured.

**Supplementary table S1: Availability of ketone measurements by calendar year**

| **Calendar year** | **All included admissions** | **Above diagnostic threshold, n (%)** | **Documented but not above threshold, n (%)** | **No ketone value available, n (%)** |
| --- | --- | --- | --- | --- |
| **2012** | 16 | 10 (62.5) | 0 (0.0) | 6 (37.5) |
| **2013** | 40 | 24 (60.0) | 0 (0.0) | 16 (40.0) |
| **2014** | 41 | 17 (41.5) | 2 (4.9) | 22 (53.7) |
| **2015** | 38 | 16 (42.1) | 0 (0.0) | 22 (57.9) |
| **2016** | 35 | 20 (57.1) | 2 (5.7) | 13 (37.1) |
| **2017** | 25 | 16 (64.0) | 0 (0.0) | 9 (36.0) |
| **2018** | 21 | 11 (52.4) | 1 (4.8) | 9 (42.9) |
| **2019** | 26 | 23 (88.5) | 0 (0.0) | 3 (11.5) |
| **2020** | 18 | 15 (83.3) | 0 (0.0) | 3 (16.7) |
| **2021** | 31 | 25 (80.6) | 0 (0.0) | 6 (19.4) |
| **2022** | 15 | 13 (86.7) | 0 (0.0) | 2 (13.3) |
| **Total** | 306 | 190 (62.1) | 5 (1.6) | 111 (36.3) |

*Table S1: Percentages are calculated within each calendar year. Data from 2012 comprise August-December, and data from 2022 comprise January-August*

**Section 2: Analyses of the temporal trend in diabetic ketoacidosis admissions**

**2.1 Main analysis**

Monthly admission counts across 121 calendar months (August 2012–August 2022), including months with zero admissions, were modelled with a log link and calendar time in years.

**Supplementary table S2A: Count models for the temporal trend in admissions**

| **Model** | **Annual RR (95% CI)** | **Additional terms** | **Pearson χ²/df** | **Deviance/df** | **AIC** | **BIC** |
| --- | --- | --- | --- | --- | --- | --- |
| **Linear Poisson** | 0.931 (0.896-0.968), p<0.001 | None | 1.084 | 1.259 | 454.611 | 460.202 |
| **Linear negative binomial** | 0.931 (0.895-0.969), p<0.001 | Ancillary parameter 0.020 | 1.041 | 1.217 | 456.475 | 464.862 |
| **Quadratic Poisson** | - | Quadratic term p=0.397 | 1.090 | 1.264 | 455.901 | 464.289 |
| **Seasonally adjusted Poisson** | 0.932 (0.896–0.969), p<0.001 | Joint seasonal test p=0.357 | 1.091 | 1.263 | 456.551 | 467.734 |
| **Segmented Poisson, fixed breakpoint January 2019** | - | Level change p=0.996; slope change p=0.203 | 1.087 | 1.263 | 456.522 | 467.705 |
| **Segmented Poisson, fixed breakpoint March 2020** | - | Level change p=0.616; slope change p=0.604 | 1.096 | 1.268 | 457.090 | 468.273 |

*Supplementary table S2A: RR denotes the annual ratio of expected monthly admission counts. Residual autocorrelation: Ljung-Box p=0.543 at lag 12*

**2.2 Sensitivity analyses restricted to admissions meeting the documented ketone criterion**

**Supplementary table S2B: Sensitivity analysis restricted to admissions meeting the documented ketone criterion**

| **Model** | **Annual RR (95% CI)** | **p-value** | **Ancillary parameter** | **Pearson χ²/df** | **Deviance/df** | **AIC** | **BIC** |
| --- | --- | --- | --- | --- | --- | --- | --- |
| **Poisson** | 0.999 (0.952-1.049) | 0.973 | - | 1.208 | 1.455 | 402.015 | 407.607 |
| **Negative binomial** | 0.999 (0.947-1.054) | 0.976 | 0.149 | 0.987 | 1.242 | 401.768 | 410.155 |

*Supplementary table S2B: Interpretation considered the temporal distribution of ketone documentation (Table S1).*

**Section 3: Analyses of factors associated with recurrent diabetic ketoacidosis admissions**

**3.1 Main analysis – factors associated with multiple lifetime admissions**

The analysis was restricted to participants with diabetes diagnosed before the index admission. Logistic regression examined the binary outcome of at least two versus one documented DKA admission. Missing disability-benefit status in 15 participants was handled using multiple imputations (Section 6).

**Supplementary table S3A: Binary logistic regression of multiple lifetime admissions**

| **Characteristic or variable** | **Lifetime admissions**  **aOR (95% CI),**  **p-value** | **Complete-case analysis**  **aOR (95% CI),**  **p-value** | **Documented-ketone analysis**  **aOR (95% CI),**  **p-value** |
| --- | --- | --- | --- |
| **Participants, n** | 148 | 133 | 88 |
| **Participants with at least two documented DKA admissions, n** | 63 | 59 | 37 |
| **Female sex** | 1.549 (0.718-3.344), p=0.265 | 1.804 (0.810-4.014), p=0.148 | 1.455 (0.498-4.256), p=0.493 |
| **Age, per 10-year increase** | 0.727 (0.565-0.935), p=0.013 | 0.720 (0.554-0.935), p=0.014 | 0.513 (0.325-0.809), p=0.004 |
| **HbA1c, per 10 mmol/mol increase** | 0.988 (0.812-1.202), p=0.901 | 0.984 (0.806-1.201), p=0.873 | 0.857 (0.628-1.170), p=0.332 |
| **Previous or current psychiatric illness** | 2.827 (1.070-7.468), p=0.036 | 2.423 (0.880-6.671), p=0.087 | 3.721 (0.897-15.435), p=0.070 |
| **Suspected treatment non-adherence** | 2.040 (0.837-4.971), p=0.117 | 2.041 (0.804-5.181), p=0.134 | 4.611 (1.108-19.197), p=0.036 |
| **Previous or current alcohol or substance misuse** | 0.668 (0.238-1.875), p=0.444 | 0.587 (0.194-1.771), p=0.344 | 0.885 (0.179-4.385), p=0.881 |
| **Receipt of disability benefits** | 4.941 (1.576-15.49), p=0.006 | 4.794 (1.499-15.327), p=0.008 | 24.365 (3.209-184.986), p=0.002 |

*Supplementary table S3A: aOR, adjusted odds ratio. Primary and documented-ketone estimates were pooled across 50 imputed datasets (Section 6). The documented-ketone analysis was restricted to participants whose original index admission met the ketone criterion, while the lifetime admission outcome was unchanged. For treatment adherence, the reference category was no concern documented.*

**3.2 Supplementary analyses – Factors associated with readmissions during follow-up**

Analyses were restricted to participants with known diabetes who survived their first observed diabetic ketoacidosis admission. Cause-specific Cox regression examined time from index discharge to first readmission, and complementary negative binomial regression modelled all subsequent admissions.

In the Cox model, participants were censored at death or the end of admission ascertainment in August 2022. The negative binomial model included the logarithm of follow-up time from index discharge until death or the end of ascertainment as an offset.

Recurrence and administrative follow-up intervals were approximated using admission month and year, with adjustment for the index hospital stay. Recurrences within the same calendar month were assigned a time of 0.5 months after discharge.

**Supplementary table S3B: Secondary analyses of first and total recurrent diabetic ketoacidosis admissions**

| **Characteristic or variable** | **First readmission: Cox regression**  **aHR (95% CI), p-value** | **All readmissions: negative binomial regression**  **aRR (95 % CI), p-value** |
| --- | --- | --- |
| **Eligible participants, n** | 143 | 143 |
| **Participants contributing to model estimation, n** | 142 | 143 |
| **Recurrent admissions analysed** | 36 | 122 |
| **Follow-up used in the count model, person-years** | - | 726.6 |
| **Female sex** | 1.488 (0.730-3.034), p=0.274 | 1.547 (0.694-3.445), p=0.287 |
| **Age, per 10-year increase** | 0.829 (0.617-1.113), p=0.212 | 0.870 (0.632-1.197), p=0.393 |
| **HbA1c, per 10 mmol/mol increase** | 1.112 (0.911-1.358), p=0.298 | 1.045 (0.842-1.298), p=0.689 |
| **Previous or current psychiatric illness** | 1.323 (0.595-2.939), p=0.492 | 2.643 (1.096-6.373), p=0.030 |
| **Suspected treatment non-adherence** | 2.119 (0.935-4.802), p=0.072 | 4.402 (1.835-10.570), p<0.001 |
| **Previous or current alcohol or substance misuse** | 1.082 (0.428-2.737), p=0.868 | 0.778 (0.270–2.239), p=0.641 |
| **Documented DKA before index admission** | 3.058 (1.469-6.365), p=0.003 | 3.755 (1.790-7.877), p<0.001 |
| **Receipt of disability benefits** | 1.535 (0.523-4.501), p=0.435 | 2.298 (0.700-7.531), p=0.170 |

*Supplementary table S3B: aHR, adjusted hazard ratio; aRR, adjusted rate ratio. All eight index-admission covariates were entered simultaneously. Estimates were pooled across 49 Cox and 50 negative binomial imputations (Section 6). One participant was censored before the earliest readmission and did not contribute to Cox estimation. Prior DKA denotes documented versus not documented.*

**3.3 Sensitivity analyses**

Both models were repeated without documented prior DKA to assess dependence on potentially incomplete historical documentation. Ketone-restricted analyses required both admissions to meet the documented criterion.

**Supplementary table S3C: Sensitivity analyses of time to first recurrent DKA admission**

| **Characteristic or variable** | **Without adjustment for documented prior DKA**  **aHR (95% CI), p-value** | **Restricted to the documented ketone criterion**  **aHR (95% CI), p-value** |
| --- | --- | --- |
| **Eligible participants, n** | 143 | 88 |
| **Participants contributing to model estimation, n** | 142 | 87 |
| **First qualifying recurrent admissions, n** | 36 | 19 |
| **Female sex** | 1.495 (0.739-3.026), p=0.264 | 0.564 (0.185-1.719), p=0.314 |
| **Age, per 10-year increase** | 0.793 (0.604-1.040), p=0.093 | 0.556 (0.309-0.998), p=0.049 |
| **HbA1c, per 10 mmol/mol increase** | 1.069 (0.881-1.296), p=0.501 | 1.290 (0.949-1.755), p=0.104 |
| **Previous or current psychiatric illness** | 1.825 (0.827-4.026), p=0.136 | 0.468 (0.137-1.599), p=0.226 |
| **Suspected treatment non-adherence** | 2.216 (0.998-4.922), p=0.051 | 1.103 (0.306-3.982), p=0.881 |
| **Previous or current alcohol or substance misuse** | 0.950 (0.383-2.356), p=0.912 | 2.198 (0.618-7.821), p=0.224 |
| **Documented DKA before index admission** | Not included | 5.929 (1.995-17.624), p=0.001 |
| **Receipt of disability benefits** | 1.763 (0.597-5.211), p=0.305 | 3.256 (0.622-17.049), p=0.162 |

*Supplementary table S3C: aHR, adjusted hazard ratio. Estimates were pooled across 49 and 50 imputations respectively. Each model had one participant censored before the earliest qualifying recurrence. The ketone restriction applies to both the index admission and the recurrence outcome.*

**Supplementary table S3D: Sensitivity analyses of the rate of recurrent DKA admissions**

| **Characteristic or variable** | **Without adjustment for documented prior DKA**  **aRR (95% CI), p-value** | **Excluding outlier patient**  **aRR (95% CI), p-value** | **Restricted to strict ketone criterion**  **aRR (95% CI), p-value** |
| --- | --- | --- | --- |
| **Model** | Negative binomial | Negative binomial | Poisson with robust standard errors |
| **Participants included, n** | 143 | 142 | 88 |
| **Participants with at least one qualifying recurrence, n** | 36 | 35 | 19 |
| **Recurrent admissions analysed, n** | 122 | 99 | 34 |
| **Female sex** | 1.294 (0.554-3.023), p=0.551 | 1.293 (0.591-2.829), p=0.520 | 0.468 (0.217-1.011), p=0.053 |
| **Age, per 10-year increase** | 0.818 (0.593-1.129), p=0.223 | 0.895 (0.657-1.218), p=0.480 | 0.601 (0.407-0.886), p=0.010 |
| **HbA1c, per 10 mmol/mol increase** | 0.962 (0.769-1.203), p=0.733 | 1.126 (0.902-1.406), p=0.295 | 1.296 (1.053-1.596), p=0.014 |
| **Previous or current psychiatric illness** | 2.860 (1.106-7.392), p=0.030 | 2.867 (1.236-6.650), p=0.014 | 0.599 (0.272-1.320), p=0.203 |
| **Suspected treatment non-adherence** | 6.712 (2.728-16.510), p<0.001 | 3.375 (1.399-8.142), p=0.007 | 1.653 (0.694-3.936), p=0.256 |
| **Previous or current alcohol or substance misuse** | 0.678 (0.217-2.117), p=0.504 | 0.794 (0.288-2.187), p=0.656 | 2.142 (1.009-4.550), p=0.047 |
| **Documented DKA before index admission** | Not included | 3.482 (1.702-7.124), p<0.001 | 6.883 (3.590-13.197), p<0.001 |
| **Receipt of disability benefits** | 2.502 (0.726-8.620), p=0.146 | 2.433 (0.780-7.585), p=0.126 | 5.264 (2.045-13.551), p<0.001 |

*Supplementary table S3D: aRR, adjusted rate ratio. Estimations were pooled across 50 imputations per analysis. All models included log-transformed full available follow-up time as an offset. Poisson regression with robust standard errors was used for the ketone-restricted count analysis because the negative binomial model encountered estimation problems. The ketone-restricted analysis counted only subsequent admissions meeting the documented ketone criterion among participants whose original index admission also met that criterion.*

**3.4 Model diagnostics**

Diagnostics were assessed in the complete-case analyses. In the logistic model, variance inflation factors ranged from 1.118 to 1.321, and excluding the participant with the largest Cook’s distance did not change the direction of the associations. Joint addition of quadratic terms for age and HbA1c did not significantly improve model fit (logistic: p=0.440; negative binomial: p=0.876). The Cox model showed no evidence of non-proportional hazards in a joint test of eight covariate-by-ln(time) interactions (p=0.646). The negative binomial dispersion parameter was 1.371, with Pearson χ²/df=1.370.

**Section 4: Survival and mortality analyses**

**4.1 Survival analyses**

Sensitivity analyses used the last observed admission as the time origin, started follow-up at discharge among index-admission survivors, or applied the documented ketone criterion (Table S4A).

**Supplementary table S4A: Five-year survival estimates**

| **Analysis** | **N** | **Deaths within five years** | **Five-year survival (95% CI)** |
| --- | --- | --- | --- |
| **First observed admission, primary analysis** | 178 | 25 | 85.3% (79.0-89.8) |
| **Last observed admission** | 178 | 26 | 84.5% (77.9-89.2) |
| **Post-discharge survival** | 173 | 20 | 87.8% (81.6-92.0) |
| **Meeting the documented ketone criterion** | 114 | 13 | 87.4% (79.2-92.6) |

**4.2 Factors associated with all-cause mortality**

Sensitivity analyses used five-year follow-up, the documented ketone restriction, and stratification by microvascular complication status.

**Supplementary table S4B: Cox regression analyses of all-cause mortality**

| **Variable** | **Primary analysis, aHR (95% CI), p-value** | **Five-year analysis, aHR (95% CI), p-value** | **Documented-ketone analysis, aHR (95% CI), p-value** |
| --- | --- | --- | --- |
| **Female sex** | 0.564 (0.275-1.156), p=0.118 | 0.538 (0.222-1.302), p=0.169 | 0.399 (0.145-1.098), p=0.075 |
| **Age, per 10-year increase** | 1.874 (1.511-2.324), p<0.001 | 1.680 (1.302-2.167), p<0.001 | 1.937 (1.386-2.706), p<0.001 |
| **HbA1c, per 10 mmol/mol increase** | 0.840 (0.699-1.009), p=0.062 | 0.819 (0.652-1.029), p=0.087 | 0.800 (0.604-1.059), p=0.120 |
| **Previous or current alcohol or substance misuse** | 2.499 (1.191-5.245), p=0.015 | 3.011 (1.268-7.148), p=0.012 | 2.125 (0.639-7.065), p=0.219 |
| **At least one macrovascular disease** | 1.666 (0.698-3.979), p=0.250 | 1.470 (0.520-4.151), p=0.467 | 1.357 (0.395-4.663), p=0.628 |
| **At least one known microvascular complication** | 1.039 (0.488-2.215), p=0.920 | 0.987 (0.397-2.456), p=0.977 | 1.244 (0.346-4.466), p=0.738 |

*Supplementary table S4B: aHR denotes adjusted hazard ratio. The primary analysis included 178 participants and 36 deaths; the five-year analysis included 178 participants and 25 deaths; and the documented-ketone analysis included 114 participants and 18 deaths. At least one macrovascular disease is a composite variable consisting of cerebrovascular, coronary and peripheral artery disease. At least one microvascular complication is a composite variable consisting of retinopathy, nephropathy and neuropathy.*

Collinearity was low (VIF 1.104-1.296), and jointly adding quadratic terms for age and HbA1c did not significantly improve model fit (p=0.314). Interactions with log-transformed analysis time suggested possible non-proportional hazards for microvascular complications (p=0.043; all other p>0.174). Stratification by microvascular complication status yielded similar estimates for the remaining covariates.

**4.3 Recurrent DKA as a time-varying covariate**

DKA readmission after the index admission was modelled as a time-varying covariate. Follow-up was administratively censored at the end of admission ascertainment in August 2022. The adjusted model included age and sex at the index admission.

**Supplementary table S4C: Recurrent DKA as a time-varying predictor of mortality**

| **Model** | **HR (95% CI)** | **p-value** |
| --- | --- | --- |
| **Unadjusted** | 1.114 (0.306-4.061) | 0.870 |
| **Adjusted for age and sex** | 1.957 (0.527-7.277) | 0.316 |

*Supplementary table S4C: Both models included 178 participants and 23 deaths. Forty participants experienced at least one recurrent admission. Recurrence timing was based on calendar month; for three participants with recurrence in the same month as the index admission, recurrence time was assigned as 0.5 months after the index admission.*

**Section 5: Factors associated with biochemical normalisation and length of hospital stay**

**5.1 Time to biochemical normalisation**

Sensitivity analyses applied the documented ketone criterion and restricted admissions to 2017–2022, when outcome documentation was more complete (Tables S5A and S6).

**Supplementary table S5A: Multivariable analyses of time to biochemical normalisation**

| **Variable** | **Primary analysis, without ketone restriction**  **Ratio of expected time (95% CI), p-value** | **Documented-ketone analysis,**  **Ratio of expected time (95% CI), p-value** | **Admissions during 2017-2022,**  **Ratio of expected time (95% CI), p-value** |
| --- | --- | --- | --- |
| **Male sex** | 0.819 (0.675-0.994), p=0.043 | 0.754 (0.602-0.944), p=0.014 | 0.793 (0.611-1.030), p=0.082 |
| **Age, per 10-year increase** | 1.048 (0.980-1.119), p=0.169 | 1.022 (0.949-1.102), p=0.561 | 1.062 (0.967-1.167), p=0.207 |
| **Admission pH, per 0.10-unit increase** | 0.755 (0.709-0.804), p<0.001 | 0.759 (0.693-0.831), p<0.001 | 0.759 (0.690-0.835), p<0.001 |
| **Admission glucose, per 10 mmol/L increase** | 0.936 (0.879-0.997), p=0.042 | 0.943 (0.842-1.057), p=0.313 | 0.873 (0.806-0.946), p<0.001 |
| **Infection as precipitating cause** | 0.887 (0.746-1.055), p=0.173 | 0.998 (0.801-1.244), p=0.985 | 0.963 (0.728-1.274), p=0.792 |
| **Calendar year, per one-year increase** | 0.973 (0.949-0.997), p=0.025 | 0.973 (0.942-1.004), p=0.088 | 0.981 (0.922-1.044), p=0.540 |

*Supplementary table S5A: All models included the six listed covariates simultaneously. Reference categories were women and the remaining precipitating-cause categories, respectively. The number of admissions/participants were 279/166 in the primary analyses, 176/121 in the documented-ketone analysis, and 131/104 in the analysis restricted to 2017-2022.*

**5.2 Length of hospital stay**

Length of hospital stay was analysed among admissions ending in live discharge. Both GEE analyses used an exchangeable working correlation structure and robust standard errors, with admissions clustered by participant.

Both GEE models yielded similar coefficient estimates with exchangeable and independent working correlation structures. For biochemical normalisation, the quadratic calendar-year term was not significant (p=0.228). Collinearity was low in the length-of-stay model (VIF 1.085-1.435). Relative residual plots showed some curvature and occasional large positive residuals. For length of stay, residuals declined at high fitted values, where observations were sparse.

**Supplementary table S5B: Multivariable analyses of length of hospital stay**

| **Variable** | **Primary analysis, ratio of expected time (95% CI), p-value** | **Documented-ketone analysis, ratio of expected time (95% CI), p-value** |
| --- | --- | --- |
| **Male sex** | 0.931 (0.806-1.074), p=0.325 | 0.918 (0.765-1.101), p=0.354 |
| **Newly diagnosed vs previously known diabetes** | 2.507 (2.033-3.090), p<0.001 | 2.280 (1.793-2.901), p<0.001 |
| **Infection as precipitating cause** | 1.199 (1.005-1.433), p=0.044 | 1.220 (0.984-1.513), p=0.069 |
| **Alcohol or substance misuse** | 0.978 (0.777-1.233), p=0.855 | 1.030 (0.751-1.414), p=0.854 |
| **At least one known macrovascular disease** | 1.873 (1.189-2.941), p=0.007 | 1.792 (0.966-3.322), p=0.064 |
| **At least one known microvascular complication** | 1.156 (0.970-1.379), p=0.106 | 1.085 (0.874-1.348), p=0.460 |
| **Age, per 10-year increase** | 1.081 (1.030-1.134), p=0.002 | 1.084 (1.023-1.150), p=0.007 |
| **Admission pH, per 0.10-unit increase** | 0.841 (0.790-0.894), p<0.001 | 0.875 (0.810-0.945), p<0.001 |
| **Admission glucose, per 10 mmol/L increase** | 1.047 (0.976-1.123), p=0.201 | 1.039 (0.947-1.139), p=0.419 |
| **Calendar year, per one-year increase** | 0.963 (0.938-0.988), p=0.004 | 0.943 (0.915-0.971), p<0.001 |

*Supplementary table S5B: Ratios of expected time were estimated using generalized estimating equations with a gamma distribution and log link. All variables were entered simultaneously. The numbers of admissions/participants were 299/173 in the primary analysis and 188/129 in the documented-ketone analysis. At least one macrovascular disease is a composite variable consisting of cerebrovascular, coronary and peripheral artery disease. At least one microvascular complication is a composite variable consisting of retinopathy, nephropathy and neuropathy.*

**Section 6: Missing data across analyses**

**Supplementary table S6: Availability of data used in the analyses**

| **Analysis or data domain** | **Variable(s)** | **Missing or unavailable, n/N (%)** |
| --- | --- | --- |
| **Temporal trend** | Monthly admission counts and calendar time | 0/121 months (0.0%) |
| **Ketone documentation** | Blood or urine ketone measurements | 111/306 admissions (36.3%) |
| **Biochemical normalisation, overall** | Time to documented bicarbonate ≥18 mmol/L | 27/306 admissions (8.8%) |
| **Biochemical normalisation, 2012-2016** | Time to documented bicarbonate ≥18 mmol/L | 22/170 admissions (12.9%) |
| **Biochemical normalisation, 2017-2022** | Time to documented bicarbonate ≥18 mmol/L | 5/136 admissions (3.7%) |
| **Admission-level regression covariates** | Admission pH; admission glucose | 2/306 admissions (0.7%) for each variable |
| **Length of stay among admissions ending in live discharge** | Time to discharge | 0/301 admissions (0.0%) |
| **Documented DKA admission history: logistic regression** | Disability-benefit status | 15/148 participants (10.1%) |
| **Readmissions during follow-up: Cox and count analyses** | Disability-benefit status | 15/143 participants (10.5%) |
| **Ketone-restricted recurrence analyses** | Disability-benefit status | 10/88 participants (11.4%) |
| **Mortality** | Six model covariates, survival time and event status | 0/178 participants (0.0%) for each variable |

*Supplementary table S6: Denominators refer to the relevant analysis populations.*

Missing disability-benefit status was multiply imputed using binary logistic regression under a missing-at-random assumption. Separate imputation models were used for the logistic, count and Cox analyses, including the other covariates and relevant outcome information: the binary outcome, recurrence counts and log follow-up time, or recurrence and death indicators with cause-specific Nelson-Aalen cumulative hazards respectively. Imputation was repeated within the ketone-restricted samples. Estimates were pooled using Rubin’s rules across 50 datasets, while the unrestricted Cox analyses used 49 because one imputation remained incomplete.

**Section 7: Biochemical parameters at admission**

**Supplementary table S7: Biochemical parameters at admission**

| **Parameter** | **Pre-existing** | **New onset** | **Total** |
| --- | --- | --- | --- |
| Blood glucose (mmol/L) | 29.0 [22.5-37.0],  n=272 | 27.5 [22.9-38.3],  n=30 | 29.0 [22.7-37.2],  n=302 |
| pH | 7.21 [7.11-7.28],  n=271 | 7.28 [7.20-7.31],  n=30 | 7.22 [7.11-7.29],  n=301 |
| pO2 (kPa) | 15.1 [13.6-16.7],  n=268 | 14.6 [13.2-15.5],  n=30 | 15.0 [13.5-16.6],  n=298 |
| pCO2 (kPa) | 2.9 [1.9-3.5],  n=271 | 3 [2.3-3.6],  n=30 | 2.9 [1.9-3.5],  n=301 |
| Bicarbonate (mmol/L) | 9.0 [4.0-12.0],  n=271 | 9.1 [5.8-13.3],  n=30 | 9.0 [4.3-12.0],  n=301 |
| Base Excess (mmol/L) | -17.9 [(-24.3)-(-13.4)], n=271 | -15.7 [(-19.9)-(-10.9)], n=30 | -17.6 [(-24.0)-(-13.1)], n=301 |
| Lactate (mmol/L) | 2.4 [1.6-3.7],  n=268 | 1.2 [0.9-2.1],  n=30 | 2.3 [1.4-3.6],  n=298 |
| Ketones capillary (mmol/L) | 5.8 [4.6-6.7],  n=53 | 5.6 [5.1-6.7],  n=9 | 5.8 [4.7-6.7],  n=62 |
| Ketones urinary | 3 [3-3],  n=125 | 3 [3-3],  n=20 | 3 [3-3],  n=145 |
| Sodium (mmol/L) | 135 [132-138],  n=270 | 136 [134-138],  n=30 | 135 [132-138],  n=300 |
| Potassium (mmol/L) | 4.8 [4.5-5.5],  n=271 | 4.2 [3.8-4.7],  n=30 | 4.8 [4.4-5.3],  n=301 |
| Effective osmolality (mOsm/kg) | 298 [291-308],  n=270 | 300 [295-310],  n=30 | 298.0 [291-308],  n=300 |

*Supplementary table S7: Values are presented as median [IQR]. n=number with complete data for each variable. 3 admissions with pre-hospital cardiac arrest excluded.*
